# Intravenous and Oral Iron Strategies for Iron-Deficiency Anemia in Pregnancy: A Systematic Review of Randomized Controlled Trials From a Hematology Perspective

**DOI:** 10.7759/cureus.106291

**Published:** 2026-04-01

**Authors:** Hector I Guerra Toro, Arturo P Jaramillo, Genesis Pazmino, Valeria M Caceres

**Affiliations:** 1 General Medicine, La Pontificia Universidad Católica del Ecuador, Quito, ECU; 2 General Medicine, Universidad Estatal de Guayaquil, Guayaquil, ECU; 3 General Medicine, University of the Americas, Quito, ECU

**Keywords:** ferrous sulfate supplements, hematology and oncology, intravenous iron sucrose (ivis), iron deficiency anemia (ida), iron-deficiency anemia in pregnancy

## Abstract

Iron-deficiency anemia is the most common hematologic disorder in pregnancy. Slow iron repletion, poor gastrointestinal tolerance, and late presentation during gestation often limit the effectiveness of oral ferrous salts, which remain the traditional first-line treatment. Intravenous iron offers faster iron delivery, but its role in routine antenatal care remains uncertain, particularly regarding its long-term safety and cost-effectiveness compared to traditional oral iron treatments. This qualitative systematic review included 10 randomized controlled trials published within the last decade that enrolled pregnant women with iron-deficiency anemia or persistent iron deficiency (defined, where applicable, as ferritin <30 μg/L after approximately four weeks of oral iron therapy) and compared intravenous iron with oral iron or one intravenous formulation with another. Across the included trials, intravenous iron generally produced more rapid ferritin replenishment and, in many studies, a faster rise in hemoglobin than oral therapy. Oral ferrous preparations were associated with more gastrointestinal adverse effects; for example, in one study, gastrointestinal treatment-related events were reported in more women receiving oral ferrous sulfate versus those receiving ferric carboxymaltose. In another trial, nausea/vomiting occurred in greater number of oral-iron recipients versus intravenous ones, while constipation and epigastric discomfort were reported only in the oral group. By contrast, serious intravenous treatment-related events were uncommon in the larger trials.​ ​Smaller and medium-sized trials generally favored parenteral iron for hematologic recovery, but the largest pragmatic studies from India, Malawi, and Nigeria showed that biochemical superiority did not consistently translate into lower rates of late-pregnancy anemia or improved major maternal and neonatal outcomes. One head-to-head intravenous trial also suggested practical and hematologic advantages of ferric carboxymaltose over iron sucrose. From a hematology perspective, intravenous iron appears most useful when rapid restoration of iron stores is needed, when adherence to oral therapy is doubtful, or when little time remains before delivery. Future trials should standardize ferritin-based diagnostic criteria, clearly distinguish hematologic from obstetric endpoints, and better define which patients are most likely to derive clinically meaningful benefit from parenteral iron.

## Introduction and background

Iron-deficiency anemia (IDA) is the most common hematologic disorder encountered in pregnancy and remains a frequent reason for specialty consultation in women of reproductive age. Globally, approximately one-third of pregnant women are affected by anemia, and iron deficiency contributes substantially to that burden, particularly in settings where nutritional inadequacy, short birth spacing, parasitic disease, and inconsistent antenatal follow-up coexist [1-5]. From a hematology perspective, pregnancy-related iron deficiency is clinically important not only because hemoglobin falls but also because maternal erythropoiesis must expand while iron is simultaneously diverted toward placental growth and fetal development. When that demand is not met, the consequences can include reduced oxygen-carrying capacity, fatigue, poor functional tolerance, impaired cognition, and greater peripartum vulnerability, while the fetus may face lower birth weight and reduced neonatal iron stores [1,2].

The physiology of pregnancy helps explain why standard oral therapy is often less effective than expected in routine practice. Maternal red cell mass rises progressively, total iron requirements accelerate during the second and third trimesters, and many women present only after stores are already depleted. Even with pregnancy-associated suppression of hepcidin, the amount of elemental iron that can be absorbed from oral preparations may be insufficient to correct moderate deficiency quickly, particularly late in gestation. In addition, ferrous salts commonly cause nausea, epigastric discomfort, constipation, and poor tolerability, which undermines adherence outside controlled settings [5]. As a result, oral iron may be less effective in women who present later in gestation, remain iron deficient despite prior supplementation, or have insufficient time before delivery for gradual repletion to achieve meaningful hematologic correction.

These limitations have renewed interest in intravenous (IV) iron formulations that bypass the gastrointestinal tract and deliver larger amounts of bioavailable iron over a shorter period. Iron sucrose has long been used because of its reassuring safety profile, whereas ferric carboxymaltose and ferric derisomaltose offer the practical advantage of high single-dose administration. For hematologists, this is relevant because treatment success in pregnancy is not defined by hemoglobin alone. Restoration of storage iron is also essential. Compared with oral therapy, IV iron produced larger short-term hematologic gains in representative trials; for example, hemoglobin rose by 22 versus 12 g/L at four weeks in Bhavi and Jaju [1], while in Chawla et al. [6], ferritin increased by 332.4 versus 62.9 ng/mL at three weeks and hemoglobin by 2.7 versus 1.6 g/dL at six weeks, supporting a practical advantage when the interval to delivery is limited. This may be especially useful in women with symptomatic anemia, previous oral treatment failure, ongoing inflammation, poor adherence, or late gestational presentation [3-10]. The key question, however, is whether this biologic advantage is consistent enough across randomized trials to support broader clinical use.

The recent trial literature offers both encouragement and caution. Smaller randomized studies have repeatedly shown faster hemoglobin recovery and markedly greater ferritin repletion with IV iron sucrose or ferric carboxymaltose than with oral ferrous salts [3-6,8,10]. Bhavi and Jaju reported larger gains in hemoglobin and iron stores with IV iron sucrose than with oral ferrous fumarate after four weeks [3]. The international FERric carboxymaltose-Assessment of SAfety and efficacy in Pregnancy (FER-ASAP) trial found that ferric carboxymaltose achieved anemia correction more often and more rapidly than oral ferrous sulfate, while causing fewer gastrointestinal adverse effects and better patient-reported vitality [5]. Such findings align with daily hematology practice, where the speed of biochemical correction often matters as much as the eventual hemoglobin plateau.

Yet, larger pragmatic trials have shown that hematologic superiority does not automatically translate into major clinical benefits. In India, Neogi and colleagues randomized more than 2,000 women with moderate-to-severe anemia and found no reduction in the primary maternal composite outcome with IV iron sucrose compared with standard oral therapy [5]. Large African trials of ferric carboxymaltose reported a similar pattern: better correction of iron deficiency and IDA, but no clear or uniform benefit for late-gestation anemia prevalence, preterm birth, or birthweight, while the large Indian phase three trial did not show improvement in the primary maternal composite outcome [11,12]. Improving iron levels can be very effective, but overall pregnancy outcomes may still not change if the anemia has multiple causes, normal pregnancy-related blood dilution continues, or the results are measured late in pregnancy after other factors have already affected the outcome.

Another challenge is the clinical, methodological, and endpoint heterogeneity of the available evidence: the included trials used different hemoglobin and ferritin eligibility thresholds, enrolled women at different gestational stages, compared different IV and oral regimens, administered different cumulative doses, and assessed outcomes at different follow-up intervals. The trials varied in baseline hemoglobin thresholds, ferritin cutoffs, gestational age at enrollment, cumulative iron dose, formulation type, and follow-up duration. Most compared IV therapy with oral ferrous sulfate or ferrous fumarate, but some compared two IV preparations directly [3-12]. Accordingly, the included trials must be interpreted across distinct endpoint domains, including hematologic outcomes such as ferritin recovery and anemia correction, obstetric outcomes such as gestational age and birthweight, and patient-centered outcomes such as tolerability, convenience, and symptom improvement. Hematologists may focus on ferritin recovery, anemia correction, and avoidance of severe anemia or transfusion. Obstetric clinicians may prioritize gestational age, birthweight, and postpartum morbidity. Patients may value tolerability, convenience, and speed of symptom improvement. A qualitative systematic review is useful in this context because it allows those priorities to be interpreted side by side rather than collapsed into a single pooled effect estimate.

The therapeutic landscape has also changed over the last decade. Earlier antenatal practice relied heavily on iron sucrose, which often requires multiple visits to replete the total iron deficit. Newer formulations, particularly ferric carboxymaltose and ferric derisomaltose, can deliver larger replacement doses in a single encounter, which may be highly relevant in busy clinics and low-resource settings [5,9,11,12]. Even so, convenience alone should not determine treatment selection. A hematology-guided approach still must weigh the severity of anemia, certainty that iron deficiency is truly present, infusion logistics, risk of adverse reactions, and the amount of time remaining before delivery.

Accordingly, the present systematic review synthesizes randomized evidence from the last 10 years on IV and oral iron strategies for pregnancy-related iron deficiency, with explicit emphasis on hematologic endpoints and clinical interpretation. The review addresses three key clinical questions: whether IV iron produces a faster or more reliable hemoglobin response than oral therapy, whether iron-store recovery consistently favors parenteral treatment, and whether those benefits are accompanied by acceptable tolerability and meaningful maternal or neonatal advantage.

## Review

Study design

This manuscript was developed as a qualitative systematic review of randomized trials evaluating therapeutic iron strategies in pregnancy and was reported in accordance with the Preferred Reporting Items for Systematic reviews and Meta-Analyses (PRISMA) 2020 framework [12]. Quantitative pooling was not undertaken because the included studies showed substantial clinical, methodological, and endpoint heterogeneity, including differences in hemoglobin and ferritin eligibility thresholds, gestational age at enrollment, IV formulation, cumulative iron dose, oral comparator regimen, follow-up interval, and the outcomes prioritized for analysis. This review was not prospectively registered in International Prospective Register of Systematic Reviews (PROSPERO). It was conducted as a focused qualitative systematic review organized around the 10 eligible full-text randomized trials retained for synthesis, and no meta-analysis was planned because of the narrative design and the substantial heterogeneity across studies.

Information sources and search approach

PubMed, Embase, and the Cochrane Library were searched for eligible studies. The search was limited to studies published within the last 10 years to reflect the contemporary therapeutic era of antenatal iron replacement. All database searches were last run on February 2, 2026. Search strategies were adapted to the syntax of each database and combined controlled vocabulary terms with free-text keywords using Boolean operators. In PubMed, MeSH terms such as “Anemia, Iron-Deficiency” and “Pregnancy” were combined with free-text terms including iron deficiency anemia, pregnancy, intravenous iron, oral iron, iron sucrose, ferric carboxymaltose, and ferric derisomaltose, using AND and OR, with exclusion of animal-only records using NOT. PubMed also applied a randomized-trial filter. Embase searches used corresponding Emtree terms and title/abstract/keyword fields, whereas CENTRAL searches used MeSH descriptors and keyword combinations within the trials register.

Eligibility criteria

Studies were eligible if they were randomized controlled trials published within the last 10 years, a time window selected to capture the contemporary era of antenatal iron therapy, including newer IV formulations such as ferric carboxymaltose and ferric derisomaltose and modern high-dose replacement strategies. Eligible trials enrolled pregnant participants with IDA or persistent iron deficiency, defined according to each study’s prespecified hematologic criteria, most commonly low ferritin; where explicitly operationalized, persistent iron deficiency referred to ferritin <30 μg/L after prior oral iron therapy. Because transferrin saturation (TSAT) was not reported uniformly across the included trials, it was not used as a mandatory review-level eligibility threshold, although it was extracted descriptively when available. Differences in iron formulation, cumulative replacement dose, number of infusions, and oral elemental iron regimen were recorded during data extraction because they were clinically meaningful and contributed to between-study heterogeneity. Non-randomized studies, postpartum-only trials, non-English reports, and studies without extractable clinical or hematologic outcomes were excluded.

Study selection and data extraction

Study selection proceeded in two stages: title and abstract screening, followed by full-text eligibility assessment. Three reviewers screened records independently at each stage using the prespecified eligibility criteria. Any disagreements regarding eligibility were resolved through consensus discussion. Data extraction was performed independently by the three reviewers using a standardized extraction form. Extracted variables included country, sample size, gestational age, hematologic entry criteria, iron formulation, comparator regimen, cumulative dose where available, duration of follow-up, hemoglobin response, ferritin or iron-store response, adverse effects, and reported maternal or neonatal outcomes. Any disagreements in extracted data were resolved through consensus review of the full text and supplementary material.

Risk of bias assessment

Risk of bias was assessed using the original Cochrane domain-based Risk of Bias framework [11]. The domains evaluated included random sequence generation, allocation concealment, blinding, incomplete outcome data, selective reporting, and other potential sources of bias. Two reviewers performed the assessment independently, and each domain was judged as low risk, high risk, or unclear risk of bias. Any disagreements were resolved through consensus discussion. Because many included trials were open-label, several studies were judged to have at least some concern for performance bias, even when laboratory outcomes were relatively objective.

Results

Literature Search and Study Selection

**Figure 1:**
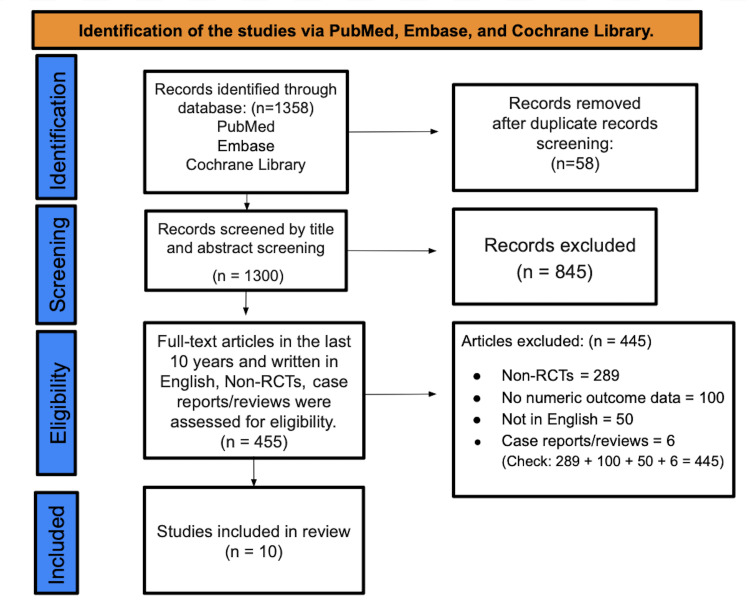
PRISMA flow diagram used for literature search and study selection PRISMA: Preferred Reporting Items for Systematic reviews and Meta-Analyses.

A total of 1,358 records were identified from PubMed, Embase, and Cochrane. Following the elimination of 58 duplicates, 1300 entries were subjected to title and abstract screening, resulting in the exclusion of 845 records. A total of 455 full-text papers were evaluated based on predetermined criteria: publication within the last decade, English language, randomized controlled design, and availability of extractable outcome data. 445 complete texts were excluded: 289 non-randomized controlled trials, 100 without extractable quantitative results, 50 in non-English languages, and six case reports or reviews. In conclusion, 10 papers fulfilled all criteria and were included in the qualitative synthesis (Figure 1) [12].

This systematic review was executed and documented in compliance with the PRISMA 2020 standards. Ten randomized trials met the eligibility criteria and were included in the final qualitative synthesis. As directed by the project materials, the study-selection workflow is displayed using the Figure 1.

Study Characteristics

The 10 included trials were published between 2017 and 2024 and together randomized 5,311 pregnant participants. Most studies compared IV iron with oral ferrous sulfate or ferrous fumarate, whereas one trial compared ferric carboxymaltose with IV iron sucrose. Trial settings ranged from single-center hospital studies to large multicenter pragmatic trials in India, Malawi, and Nigeria. Follow-up periods ranged from four weeks to delivery and early postpartum follow-up. The dominant outcomes were hemoglobin change, ferritin or iron-store recovery, anemia correction, tolerability, and selected maternal or neonatal endpoints (Table 1).

**Table 1:** Study characteristics of the included randomized trials RCT: randomized clinical trials; FMC: Ferric Carboxymaltose; Hb: hemoglobin; IV: intravenous.

Study	Setting / sample	Intervention	Comparator	Follow-up	Key hematology findings
Bhavi et al., 2017 [1]	India; n=112; prospective RCT	IV iron sucrose 200 mg/day to calculated dose	Oral ferrous fumarate 200 mg/day	4 weeks	Greater Hb rise and markedly larger ferritin increase with IV iron; fewer gastrointestinal adverse effects.
Ruangvutilert et al., 2017 [2]	Thailand; n=80; RCT	Weekly IV iron sucrose 200 mg up to 500 mg	Daily oral iron (ferrous fumarate)	Until delivery	The late-pregnancy IV strategy was designed for faster correction with lower pill burden; hematologic efficacy favored parenteral treatment.
Breymann et al., 2017 [3]	Multinational; n=252; open-label RCT	Ferric carboxymaltose 1000-1500 mg	Oral ferrous sulfate 200 mg/day	12 weeks	Higher and faster anemia correction with FCM, less gastrointestinal toxicity, and better vitality/social functioning.
Jose et al., 2019 [4]	India; n=100; open-label RCT	Ferric carboxymaltose	IV iron sucrose complex	12 weeks	FCM achieved faster repletion and required fewer visits than iron sucrose, with favorable hematologic recovery.
Neogi et al., 2019 [5]	India; n=2018; multicenter phase 3 RCT	Calculated-dose IV iron sucrose	Standard oral elemental iron 100 mg twice daily	Through delivery and postpartum	Biologically effective but did not reduce the primary maternal composite outcome; serious adverse events were not treatment-related.
Chawla et al., 2022 [6]	India; n=362; randomized trial	Single-dose ferric carboxymaltose 1000 mg	Oral ferrous sulfate 120 mg/day	6 weeks	Larger Hb and ferritin rise with FCM, fewer gastrointestinal complaints, and no clear neonatal difference.
Hansen et al., 2023 [7]	Denmark; n=201; single-center RCT	Ferric derisomaltose 1000 mg single dose	Oral ferrous fumarate 100 mg/day	18 weeks	More women remained non-anemic; IV therapy improved Hb, fatigue, and quality of life while maintaining comparable safety.
Chauhan et al., 2023 [8]	India; n=268; open-label RCT	IV iron sucrose	Oral ferrous sulfate	Short-term follow-up	Quicker Hb and ferritin response with IV iron and better tolerability in a late-presenting population.
Pasricha et al., 2023 [9]	Malawi; n=862; open-label RCT	Ferric carboxymaltose up to 1000 mg once	Standard oral elemental iron for 90 days	36 weeks plus postpartum	No difference in 36-week anemia or birthweight, but earlier hematologic improvement and good safety profile.
Afolabi et al., 2024 [10]	Nigeria; n=1056; multicenter open-label RCT	Single-dose ferric carboxymaltose 20 mg/kg (max 1000 mg)	Oral ferrous sulfate until 6 weeks postpartum	36 weeks plus postpartum	No significant difference in anemia at 36 weeks or preterm birth, but lower iron deficiency and iron-deficiency anemia with IV treatment.

Risk of Bias

Most studies were randomized and clinically relevant, but open-label treatment was common. As a result, the structured summary was characterized by some concerns rather than low risk of bias. Objective laboratory outcomes reduced detection bias in several trials, whereas concealment procedures were not always described in detail (Table 2) [11].

**Table 2:** Qualitative risk of bias assessment of the included trials

Study	Randomization / concealment	Blinding / deviations	Outcome data / reporting	Overall judgment	Concise rationale
Bhavi et al., 2017 [1]	Some concerns	Some concerns	Low	Some concerns	Small open-label trial; objective hematologic outcomes reduce detection bias, but allocation detail was limited.
Ruangvutilert et al., 2017 [2]	Some concerns	High	Low	Some concerns	A randomized design was reported, but masking was not evident, and the accessible report provided limited methodological detail.
Breymann et al., 2017 [3]	Low	Some concerns	Low	Some concerns	Large randomized international trial; open-label design introduces potential performance bias despite objective outcomes.
Jose et al., 2019 [4]	Low	High	Low	Some concerns	Computer-generated block randomization was described, but the trial was open-label.
Neogi et al., 2019 [5]	Low	High	Low	Some concerns	Large multicenter phase 3 trial with robust reporting; open-label treatment remained the main concern.
Chawla et al., 2022 [6]	Some concerns	High	Some concerns	Some concerns	Randomized but analyzed per protocol, with losses to follow-up and open-label treatment.
Hansen et al., 2023 [7]	Low	High	Low	Some concerns	Web-based randomization and structured follow-up were strengths; open-label delivery remained unavoidable.
Chauhan et al., 2023 [8]	Some concerns	High	Low	Some concerns	Open-label trial with limited detail on concealment, but clinically relevant outcomes were clearly reported.
Pasricha et al., 2023 [9]	Low	Some concerns	Low	Some concerns	Randomized with masked laboratory/birthweight assessment; participants and nurses were not masked.
Afolabi et al., 2024 [10]	Low	Some concerns	Low	Some concerns	Large web-randomized pragmatic trial with comprehensive outcome reporting; primary unmasking created some concern for deviations.

Qualitative Evidence Synthesis

Hemoglobin response: Across the review, the most reproducible hematologic finding was a faster early hemoglobin response with IV iron. Bhavi and Jaju [1] reported a greater four-week hemoglobin increment with IV iron sucrose than with oral ferrous fumarate (22 ± 11.5 g/L vs 12 ± 9 g/L), and a hemoglobin rise greater than 20 g/L occurred in 55% versus 11% of participants, respectively. Breymann and colleagues [3] reported that ferric carboxymaltose achieved anemia correction more often than oral ferrous sulfate (84% vs 70%) and in a shorter median time (3.4 vs 4.3 weeks); by week six, the mean hemoglobin increase was also greater with ferric carboxymaltose (1.75 ± 1.18 g/dL vs 1.32 ± 1.54 g/dL). Chawla et al., Hansen et al., and Chauhan et al. [6-8] likewise reported quicker hemoglobin improvement with parenteral therapy. In contrast, the larger pragmatic studies showed a more nuanced picture. Neogi et al. [5] demonstrated that IV iron sucrose was biologically active but did not improve the prespecified maternal composite outcome, and the Malawian and Nigerian ferric carboxymaltose trials did not show a decisive reduction in late-pregnancy anemia prevalence despite favorable intermediate responses [9,10]. Collectively, these findings suggest that IV iron has a clear hematologic advantage for early correction, but that the magnitude of the benefit depends on baseline severity, gestational timing, and the endpoint chosen.

Iron stores and ferritin recovery: Serum ferritin recovery and trial-defined correction of iron deficiency favored IV therapy more consistently than hemoglobin did. This pattern was seen in the small iron sucrose trials, in the ferric carboxymaltose studies, and in the ferric derisomaltose trial. From a hematology standpoint, this is highly relevant because ferritin recovery reflected materially greater restoration of storage iron in representative trials; for example, Bhavi and Jaju [1] reported a four-week ferritin increase of 112.17 ± 98.15 ng/mL versus 22.71 ± 11.32 ng/mL with IV versus oral therapy, and Jose et al. [4] found a three-week median ferritin of 343 versus 298 μg/L with ferric carboxymaltose versus iron sucrose. Bhavi and Jaju [1] reported markedly greater ferritin improvement with IV iron sucrose. Jose et al. [4] showed that ferric carboxymaltose replenished stores more quickly than iron sucrose while requiring fewer visits. The Intravenous versus oral iron for iron deficiency anaemia in pregnant Nigerian women (IVON) trial [10] demonstrated a substantially lower prevalence of iron deficiency and iron-deficiency anemia at 36 weeks among women treated with ferric carboxymaltose, even though overall anemia prevalence remained similar between groups. These data suggest that parenteral iron is particularly effective when the clinical objective is complete repletion of iron deficit rather than partial symptomatic improvement.

Tolerability and safety: A second consistent signal was the poorer tolerability of oral ferrous preparations. In the FER-ASAP trial [3], treatment-related adverse events occurred in 14 women (11%) receiving ferric carboxymaltose versus 19 women (15%) receiving oral ferrous sulfate, with gastrointestinal disorders reported in three versus 16 women, respectively. In Chawla et al. [6], nausea/vomiting occurred in five versus 31 women, constipation in zero versus 27, and epigastric discomfort in zero versus 23 in the IV versus oral groups. Chauhan et al. [8] likewise reported fewer adverse effects with IV iron sucrose, with eight mild hypersensitivity-type events in the IV group compared with 51 adverse-effect reports in the oral group. Serious infusion-related toxicity remained uncommon. In Neogi et al. [5], maternal serious adverse events occurred in 16 women receiving IV iron sucrose and 13 receiving oral therapy, while serious fetal/neonatal adverse events occurred in 39/961 (4%) versus 45/982 (5%), and none was considered treatment-related. Similarly, the Malawian ferric carboxymaltose trial found no infusion-related serious adverse events and no significant difference in overall adverse events (43% vs 39%) [9], and the Nigerian IVON trial [10] likewise reported no significant difference in adverse events between groups. Thus, the classic concern that IV iron is inherently more hazardous than oral treatment was not strongly supported in these contemporary pregnancy trials, although infusion monitoring and formulation-specific precautions remain appropriate.

Maternal and neonatal outcomes

When maternal and neonatal outcomes beyond laboratory correction were examined, the evidence was less consistent, particularly for the primary maternal composite outcome, anemia prevalence at 36 weeks, birthweight, and preterm birth. The Indian phase three trial did not show benefit of IV iron on the primary maternal composite outcome [5]. The Malawian trial found no difference in birthweight and no significant reduction in anemia prevalence at 36 weeks, despite early post-treatment gains and reassuring safety [9]. Similarly, the Nigerian IVON study [10] found no significant difference in maternal anemia at 36 weeks or preterm birth, but it did show better correction of iron deficiency and iron-deficiency anemia. Most smaller trials were not powered for obstetric endpoints and therefore mainly inform hematologic rather than perinatal decision-making. This distinction is important: failure to change birthweight or preterm birth does not negate the hematologic value of restoring iron stores, but it does argue against assuming that every laboratory advantage will translate into a measurable obstetric benefit.

Comparative IV formulations

The single head-to-head randomized trial comparing ferric carboxymaltose with IV iron sucrose adds a practical dimension to the literature. Jose et al. [4] reported more rapid repletion and fewer treatment visits with ferric carboxymaltose. Hematologically, this suggests that formulation choice is not trivial. The ability to replace a substantial iron deficit in one or two sessions may improve treatment completion, reduce missed appointments, and accelerate store recovery in women presenting late in pregnancy. For clinicians managing antenatal anemia services, convenience therefore intersects with effectiveness.

Discussion

Across the included randomized trials, IV iron generally produced faster ferritin recovery and, in many studies, a more rapid hemoglobin response than oral therapy, with fewer gastrointestinal adverse effects; however, the largest pragmatic trials did not show clear improvement in the primary maternal composite outcome, anemia prevalence at 36 weeks, birthweight, or preterm birth, despite better correction of iron deficiency and IDA [1-10]. Although oral iron continues to be the usual first-line treatment in many settings, the randomized controlled trials included in this review suggest that IV iron often leads to a quicker rise in hemoglobin and better replenishment of iron stores than oral therapy [1-10]. This pattern becomes especially important in pregnancy because untreated or poorly treated anemia can affect both maternal well-being and fetal outcomes, and the time available to correct anemia may be limited depending on gestational age [13-18].

The earlier trials in this review already pointed in this direction. Bhavi and Jaju found that IV iron sucrose was more effective than oral ferrous fumarate in treating anemia in pregnancy, showing that IV therapy can be a practical and successful option in women who need stronger correction than oral treatment may provide [1]. In the same way, Ruangvutilert and colleagues compared weekly low-dose IV iron sucrose with daily oral iron in women with IDA during late pregnancy and also reported favorable outcomes with IV treatment [2]. This is especially meaningful because women who present in late pregnancy have less time before delivery, so a faster treatment option may be more clinically useful [2]. These early studies suggest that IV iron should not only be viewed as a backup treatment, but in some cases as a preferred option depending on the patient’s condition and timing in pregnancy [1,2].

The studies involving ferric carboxymaltose expanded the contemporary evidence base on IV iron, particularly with respect to ferritin recovery, hemoglobin response, and treatment practicality. In the FER-ASAP trial, Breymann and colleagues showed that ferric carboxymaltose was effective compared with oral iron and had the added advantage of allowing larger doses to be administered in fewer visits [3]. That practical benefit matters in prenatal care because repeated hospital or clinic visits may be difficult for pregnant women due to transportation, work, childcare, or other barriers. Jose and colleagues compared ferric carboxymaltose with iron sucrose complex and found that both IV treatments were effective in improving IDA during pregnancy [4]. Their findings are useful because they show that the benefits of IV therapy are not limited to just one formulation. Chawla and colleagues later compared ferric carboxymaltose with oral iron and again reported positive results for the IV group, adding more support to the idea that modern IV formulations may be particularly helpful when rapid iron replacement is needed [6].

Among the 10 studies, some of the strongest evidence came from the larger and more recent multicenter trials. Neogi and colleagues conducted a phase three randomized controlled trial in India and showed that IV iron sucrose was both safe and effective in pregnant women with moderate-to-severe anemia [5]. This study is especially important because it focused on women with more serious anemia, which is often the group most likely to benefit from faster and more reliable treatment. In these patients, waiting for oral iron to work may not be ideal, especially if the patient is symptomatic or approaching delivery [5]. Pasricha and colleagues also contributed major evidence through their trial in Malawian women with second-trimester anemia, where ferric carboxymaltose was compared with standard oral care [9]. Their study showed that IV iron can be effective even in lower-resource settings with a high burden of maternal anemia, which expands the relevance of this treatment beyond high-income countries [9]. Similarly, Afolabi and colleagues in the IVON trial showed that IV iron could be successfully used in pregnant women in Nigeria, again supporting its usefulness in settings where anemia is common and where an effective intervention may have important public health value [10].

The studies from 2023 also added valuable detail to the overall picture. Hansen and colleagues compared ferric derisomaltose with oral iron in pregnant women with persistent iron deficiency and found that IV treatment remained beneficial with newer iron formulations as well [7]. Chauhan and colleagues compared iron sucrose with ferrous sulfate and also found results that favored IV treatment [8]. Together, these trials show that the advantage of IV iron has remained consistent across different years, settings, and formulations. They also suggest that the question is no longer simply whether IV iron works in pregnancy, but rather which patients will benefit the most, which preparation is most practical, and at what stage of pregnancy IV therapy should be considered [7-10].

Even though the overall pattern supports IV iron, the results were not exactly the same across all trials, and this should be acknowledged. The included studies varied in several important ways, including gestational age at enrollment, baseline severity of anemia, oral comparator used, IV formulation, and dosing schedule [1-10]. Some studies included women later in pregnancy, where time to delivery was short, while others enrolled women earlier, allowing oral therapy more time to show a benefit [2,9]. Some trials focused on moderate-to-severe anemia, while others included broader patient groups [5,10]. These differences likely explain why the size of the treatment effect was not identical in every study. Still, despite this heterogeneity, the direction of the evidence was largely the same, with IV iron generally producing faster and stronger hematologic improvement than oral iron [1-10].

Safety is also an important consideration. Based on the trials included in this review, IV iron was generally well tolerated and did not show major safety concerns that outweighed its clinical benefits when used appropriately [3-10]. At the same time, IV treatment does require more medical resources, including infusion facilities, observation, and trained staff, which may limit its accessibility in some settings. Oral iron, despite its slower effect and frequent gastrointestinal side effects, remains easier to prescribe, less resource-intensive, and more widely available [13-15]. For that reason, it would not be accurate to say that IV iron should replace oral iron in every pregnant patient. Instead, the evidence suggests that IV iron may be especially useful for women with moderate-to-severe anemia, poor response to oral therapy, intolerance to oral iron, or limited time before delivery [13-18].

The broader secondary literature supports this interpretation. Reviews, meta-analyses, and guideline papers generally conclude that IV iron is associated with a faster increase in hemoglobin and better correction of iron deficiency than oral therapy, although they also emphasize that treatment decisions should be individualized based on severity, timing, and available resources [16-27]. This is consistent with a hematology-focused perspective, where treatment choice should depend not only on whether anemia is present, but also on how severe it is, how quickly it needs to be corrected, and whether oral therapy is realistic for that patient [16,17]. Current guidelines also continue to support oral iron as a reasonable first step in many uncomplicated cases, while recognizing IV iron as an important option when oral therapy is not sufficient or not tolerated [13-15].

Overall, the 10 randomized controlled trials included in this review support a hematologic advantage of IV iron in pregnancy, particularly for faster ferritin repletion and, in many studies, more rapid hemoglobin improvement, especially in women who require prompt correction or do not tolerate oral therapy well [1-10]. Although more research is still needed on formulation-specific comparisons, cost-effectiveness, and long-term maternal and neonatal outcomes, the current evidence already supports a meaningful role for IV iron in the management of IDA during pregnancy [20-27].

Limitations

Several limitations should be acknowledged. First, this review was not prospectively registered in PROSPERO, which reduces prospective methodological transparency even though the eligibility criteria and qualitative synthesis objectives were defined during manuscript preparation. Second, the review was restricted to 10 randomized trials and did not include meta-analyses, so the findings should be interpreted as a structured qualitative synthesis rather than a pooled quantitative estimate. Third, the included studies showed substantial clinical and methodological heterogeneity, including differences in baseline hemoglobin thresholds, ferritin cutoffs, gestational age at enrollment, IV formulation, cumulative iron dose, comparator regimen, follow-up duration, and prioritized endpoints. Fourth, many of the included trials were open label, and several were single-center studies, which increases the possibility of performance bias and limits generalizability despite the relative objectivity of laboratory outcomes. Fifth, outcome definitions were not fully standardized across studies, particularly for anemia correction, iron deficiency, maternal composite outcomes, and neonatal endpoints, which limited direct between-study comparison. Finally, the study populations differed in baseline anemia severity, iron status, malaria exposure, and concomitant antenatal interventions, factors that may have influenced both hematologic and obstetric outcomes. Despite these limitations, the overall direction of the hematologic evidence was broadly similar across trials, especially with respect to faster ferritin recovery and better tolerability of IV therapy relative to oral iron.

## Conclusions

IV iron provides a reliable hematologic advantage in pregnancy, with consistently faster ferritin repletion and usually more rapid hemoglobin improvement than oral ferrous salts, while oral therapy is frequently limited by gastrointestinal intolerance and slower repletion. 

However, the larger contemporary pragmatic trials indicate that biochemical superiority does not consistently translate into improved major obstetric outcomes, supporting a targeted approach in which IV iron is prioritized for late gestation, poor oral tolerance, suspected nonadherence, or when rapid restoration of iron stores is clinically necessary.
